# Autoimmunity in monogenic combined immune deficiencies with associated or syndromic features

**DOI:** 10.3389/fimmu.2022.1023127

**Published:** 2022-12-05

**Authors:** Niusha Sharifinejad, Gholamreza Azizi, Zahra Chavoshzadeh, Seyed Alireza Mahdaviani, Mahnaz Seifi Alan, Marzieh Tavakol, Homa Sadri, Mohammad Nabavi, Sareh Sadat Ebrahimi, Afshin Shirkani, Ahmad Vosughi Motlagh, Molood Safarirad, Fatemeh Aghamahdi, Farzad Nazari, Samaneh Delavari, Mahnaz Jamee, Farimah Fayyaz, Parham Samimisedeh, Rahman Matani, Marzie Esmaeili, Reza Yazdani, Nima Rezaei, Hassan Abolhassani

**Affiliations:** ^1^ Non-communicable Diseases Research Center, Alborz University of Medical Sciences, Karaj, Iran; ^2^ Research Center for Immunodeficiencies, Pediatrics Center of Excellence, Children’s Medical Center, Tehran University of Medical Sciences, Tehran, Iran; ^3^ Pediatric Infections Research Center, Mofid Children’s Hospital, Shahid Beheshti University of Medical Sciences, Tehran, Iran; ^4^ Pediatric Respiratory Diseases Research Center, National Research Institute of Tuberculosis and Lung Diseases, Shahid Beheshti University of Medical Sciences, Tehran, Iran; ^5^ Cardiovascular Research Center, Alborz University of Medical Sciences, Karaj, Iran; ^6^ Department of Allergy and Clinical Immunology, Rasool e Akram Hospital, Iran University of Medical Sciences, Tehran, Iran; ^7^ Department of Immunology and Allergy, Kerman University of Medical Sciences, Kerman, Iran; ^8^ Allergy and Clinical Immunology Department, School of Medicine, Bushehr University of Medical Science, Bushehr, Iran; ^9^ Department of Pediatrics, North Khorasan University of Medical Sciences, Bojnurd, Iran; ^10^ Pediatric Nephrology Research Center, Research Institute for Children’s Health, Shahid Beheshti University of Medical Sciences, Tehran, Iran; ^11^ Cancer Immunology Project (CIP), Universal Scientific Education and Research Network (USERN), Tehran, Iran; ^12^ Division of Clinical Immunology, Department of Biosciences and Nutrition, Karolinska Institutet, Karolinska University Hospital, Huddinge, Stockholm, Sweden

**Keywords:** inborn errors of immunity, primary immunodeficiency, combined immunodeficiency syndrome, autoimmunity, immune dysregulation

## Abstract

**Background:**

Combined immune deficiencies (CIDs) with associated or syndromic features are a highly heterogeneous subgroup of inherited immune disorders. These patients represent specific clinical complications with an increased risk of autoimmune conditions.

**Methods:**

We analyzed data of monogenic patients with syndromic CIDs adopted from the Iranian inborn errors of immunity registry up to January 2022. A comprehensive comparison in terms of demographic, clinical, and immunological features was performed between patients with and without autoimmunity and also among four mutation groups with the most registered cases including *ATM*, *STAT3 (AD-LOF)*, *DNMT3B/ZBTB24*, and *WAS* mutations.

**Results:**

A total of 137 patients with monogenic syndromic CIDs were included. Most commonly mutated genes were the *ATM* [80 (58.4%)] and *STAT3 (AD-LOF)* [19 (13.9%)], followed by *DNMT3B* [11 (8%)], and *WAS* [11 (8%)]. More than 18% of all patients with syndromic CIDs, including most *DNMT3B/ZBTB24* mutations patients, were clinically diagnosed with antibody deficiencies before genetic evaluation. Patients with *ATM* and *WAS* mutations had the latest age of onset and the lowest age of diagnosis, respectively. Autoimmune disorders were diagnosed in 24 patients at a median age of 3.5 (2.6-6.0) years, 70.6% of which were diagnosed prior to the diagnosis of immunodeficiency. Lymphoproliferation, particularly hepatosplenomegaly, was significantly higher in patients with autoimmunity (*p=0.004*). Syndromic CID patients with autoimmunity had significantly lower IgG levels. Hematologic autoimmunity mainly immune thrombocytopenic purpura was the most frequent autoimmunity among major groups of *ATM*, *STAT3 (AD-LOF)*, *DNMT3B/ZBTB24*, and *WAS* mutations, however *ATM-*mutated patients present more diversified involved organs including rheumatologic, gastrointestinal and dermatologic autoimmunity.

**Conclusion:**

About 18% of patients with monogenic syndromic CIDs developed autoimmunity, mainly in the form of hematological immune diseases. Autoimmunity could be an early-onset involvement with a potential diagnostic impact on suspicious cases of syndromic CIDs.

## Introduction

Combined immune deficiencies (CIDs) with associated or syndromic features are a highly heterogeneous subgroup of inborn errors of immunity (IEIs) affecting the development and/or function of adaptive immunity mainly T lymphocytes, with variable non-immunologic defects due to extrinsic function of the defective genes in both immune system and other human organs (1). These CIDs are classified into eight categories: congenital thrombocytopenia, syndromic DNA repair defects, immunoosseous dysplasias (IODs), thymic defects, hyper-immunoglobulin E syndromes (HIES), defects of vitamin B12 and folate metabolism, anhidrotic epidermodysplasia with immunodeficiency (EDA-ID), and others syndromic CIDs (2, 3). Patients suffering from syndromic CIDs manifest heterogeneity at the molecular level, explaining characteristic extra-immune clinical symptoms, incomplete penetrance, and variable expressivity in many associated diseases (4). The majority of CIDs with syndromic features are monogenic diseases caused by point mutations with few cases of chromosomal aberrations (e.g. syndromic DNA repair defects with DiGeorge/velocardiofacial syndrome). Of note, gain- or loss-of-function mutations in some distinct genes determine the type of immune defect in the patients. In *WAS* gene, for example, loss-of-function (LOF) mutations cause the Wiskott-Aldrich syndrome, while *WAS* gain-of-function (GOF) mutations lead to X-linked neutropenia with a different prognosis and therapeutic approach (2, 3).

Most patients with syndromic CIDs represent specific clinical manifestations, other than expected CID related infectious complications, in correlation with the type of their mutation (4). Generally, CID patients show a range of clinical involvements counting autoimmunity as a well-recognized presentation (5, 6). An increased risk of autoimmune conditions as well as certain malignancies has been reported in syndromic CIDs (4, 7). Some organ-specific autoimmune disorders were observed in these patients including hematologic autoimmunity in patients with *PNP, STIM1, ORAI1, WAS, ATM*, and *STAT5B* mutations or early-onset inflammatory bowel disease in patients with *WAS*, *DNMT3B*, and *ZBTB24* mutations (4). Despite former studies about autoimmunity in IEI, the information regarding syndromic CIDs is quite rare (8, 9). Although autoimmune disorders often take time to develop, they may be the predominant or initial manifestation of syndromic CIDs. Therefore, increasing awareness regarding the spectrum of autoimmune disorders in syndromic CIDs can promote clinicians to identify an underlying genetic defect after detecting organ-specific autoimmune abnormalities and closely monitor certain organs after establishing a specific monogenic immunodeficiency diagnosis.

In this regard, we conducted a study on a considerable sample size of 137 monogenic patients to elucidate the prevalence and spectrum of autoimmune disorders among Iranian monogenic patients with syndromic CID.

## Patients and method

### Study population

This retrospective cross-sectional study was conducted on monogenic patients with syndromic CID enrolled in the Iranian IRI registry up to January 2022. To select eligible patients, the list of responsible genes for syndromic CIDs was extracted from the 2019 update of the International Union of Immunological Societies (IUIS) (2), which was available at the time, and was investigated in the national registry. The database is hosted in the Research Center for Immunodeficiencies, Children’s Medical Center (Tehran, Iran) which provides a referral center for suspected or diagnosed IEI cases from all over Iran. This study was approved by the Ethics Committee of the National Institute for Medical Research Development (IR.NIMAD.REC.1400.086). Prior to data collection, written informed consent has been obtained from each patient and/or their parents.

### Data collection

The clinical diagnosis of CID was made according to the criteria of the European Society for immunodeficiencies (ESID) (10). A proper questionnaire surveyed the patients’ demographic information including sex, age of disease onset, age of diagnosis, family history, detailed clinical history including autoimmunity, previous infections, non-immune manifestations and laboratory data. The evaluation for autoimmunity was reviewed for all patients by an Immunologist and a subspecialist related to the affected organ. The presence of more than one autoimmune disease in a single patient was defined as polyautoimmunity. Secondary defects of the immune system were excluded. Laboratory evaluations were performed as indicated, including complete blood and differential counts, serum immunoglobulin levels, disease-specific autoantibody measurements, and flow cytometric evaluation of lymphocyte subsets as described previously (11).

### Genetic analysis

The patients’ whole blood samples were used for genomic DNA extraction. Targeted or whole-exome sequencing was performed for patients depending on whether they had the classical clinical presentations suggestive of a specific IEI, using a pipeline described previously (11–14). Sanger sequencing was used to confirm the candidate pathogenic variant found in next-generation sequencing (NGS). The pathogenicity of all disease-attributable gene variants was reassessed based on the updated guideline of the American College of Medical Genetics and Genomics (ACMG) for molecular sequencing interpretation (15). Only monogenic cases were retained and patients with chromosomal aberrations were excluded from this study as several genes can be affected with these large defects.

### Statistical analysis

All of the statistical analyses were performed using SPSS version 26.0 (IBM, Chicago, IL, USA). Qualitative variables were reported by absolute numbers and percentages. For quantitative data, median and interquartile ranges (IQR) were calculated. We compared the data of patients with and without autoimmunity. Furthermore, four main groups with the most registered cases, including ataxia-telangiectasia mutated (*ATM*), autosomal dominant LOF signal transducer and activator of transcription 3 [*STAT3 (AD-LOF)*], DNA methyltransferase 3 Beta *(DNMT3B)*/Zinc finger and BTB domain containing 24 *(ZBTB24)*, and *WAS (LOF)* mutations, were compared. Wilcoxon, and Chi-square or Fisher exact tests were utilized for the comparison. Statistically significant results were considered with a p-value of less than 0.05.

## Results

### Demographic characteristics

**Figure 1:**
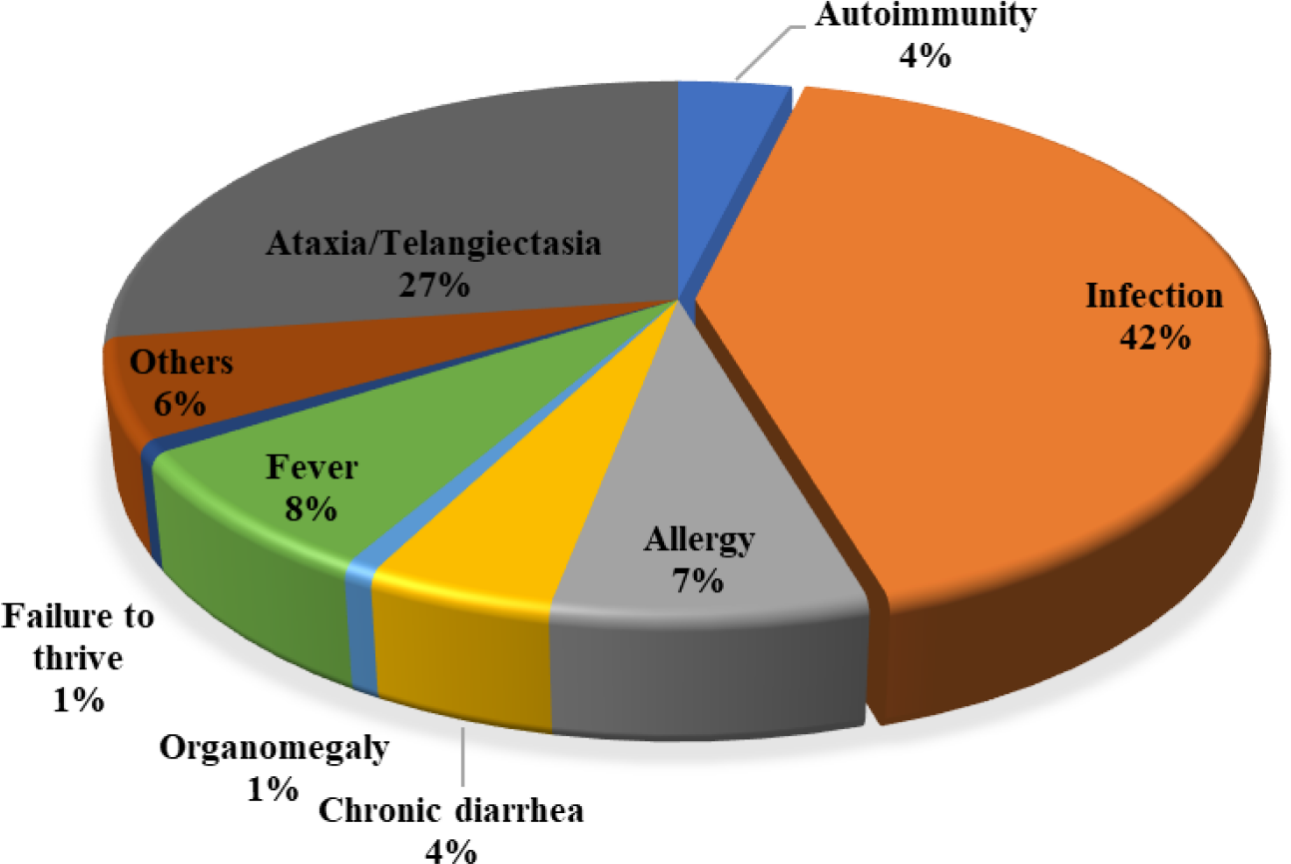
The chief complaint (at first visit) of Iranian patients with syndromic CID.

A hundred and thirty-seven patients with monogenic syndromic CIDs were included in the study. Sixty-nine individuals (50.4%) were female. The majority of patients first presented with various types of infections (55 of 132, 41.7%) and ataxia and/or telangiectasia (36 of 132, 27.3%) at a median (IQR) age of 12.0 (3.0-24.0) months (**Figure 1**, **Table 1**). More than 77% of cases with available data, mainly from patients with AR disorders, were born to consanguineous parents. The diagnosis of syndromic CID was established at a median (IQR) age of 4.0 (2.0-7.0) years with a median (IQR) diagnostic delay of 2.2 (0.5-5.0) years. Among patients with available data regarding their life status (10 patients were not available at the last visit), 30 (23.6%) patients deceased within a median (IQR) of 10 (6.3-16.1) years of follow-up. Based on ESID criteria, most patients were clinically diagnosed with ataxia-telangiectasia (55%), hyper IgE syndrome (13.1%), and antibody deficiencies (11.7%) before genetic evaluation (**Figure S1**).

**Table 1:** The demographic data of Iranian patients with syndromic CID.

Parameters	Total (N=137)	Patients with autoimmunity (N=24)	Patients without autoimmunity (N=110)	*^†^* P-value	ATM(N=80)	STAT3-AD (N=19)	DNMT3B/ZBTB24 (N=19)	WAS (N=11)	*^§^* P-value
Male/Female, (n=137)	68/69	10/14	57/53	0.367	36/44	10/9	8/11	10/1	0.126
Age of onset, months, median (IQR), (n=130)	12.0 (3.0-24.0)	12.0 (6.0-33.6)	12.0 (3.0-24.0)	0.218	12.0 (11.0-24.0)	0.2 (0-3.0)	3.0 (0-6.0)	0.1 (0-0.5)	**<0.001**
Age of autoimmunity diagnosis, years, median (IQR), (n=16)	3.5 (2.6-6.0)	3.5 (2.6-6.0)	–	–	3.0 (2.8-9.5)	3.5 (1.0-)	4.0 (2.5-6.0)	3.2 (0.5-5.5)	0.926
Age of IEI diagnosis, years, median (IQR), (n=127)	4.0 (2.0-7.0)	4.5 (3.0-7.0)	4.0 (2.0-7.0)	0.551	5.0 (2.8-7.0)	6.0 (3.0-12.0)	2.0 (0.7-4.5)	0.5 (0-6.5)	**0.006**
Delay in diagnosis, years, median (IQR), (n=128)	2.2 (0.5-5.0)	2.0 (0.1-4.0)	3.0 (1.0-5.0)	0.251	3.0 (1.0-5.0)	5.0 (1.0-11.0)	1.4 (0.1-4.6)	0.2 (0-1.0)	**0.040**
Course of disease, years, median (IQR), (n=130)	10.0 (6.3-16.1)	8.5 (3.3-11.7)	11.0 (7.0-17.0)	0.072	9.7 (7.0-14.0)	24.0 (12.0-33.0)	10.8 (2.3-21.2)	4.5 (2.0-11.5)	**<0.001**
Consanguinity, n (%), (n=134)	104 (77.6%)	19 (79.2%)	85 (73.3%)	0.840	68 (86%)	10 (52.6%)	16 (88.9%)	5 (50%)	**0.001**
Dead, n (%), (n=127)	30 (23.6%)	4 (19%)	26 (24.8%)	0.575	25 (31.6%)	2 (12.5%)	0	3 (30%)	**0.013**

n, number of patients with available data; N, total number of patients; ATM, Ataxia-telangiectasia; IEI, inborn errors of immunity.

The median is shown [with 25th and 75th percentiles].

*p-value is statistically significant <0.05 (represented in bold).

†comparing patients with or without autoimmunity.

§comparing ataxia-telangiectasia, HIES, WAS, and ICF syndrome (DNMT3B/ZBTB24 deficiencies).

### Molecular findings

Disease-causing mutations were detected in 11 unique genes through whole-exome sequencing (WES) or targeted gene sequencing (**Table S1**). Most commonly mutated genes were the *ATM* [80 (58.4%)] and *STAT3* [19 (13.9%)], followed by *DNMT3B* [11 (8%)], *WAS* [11 (8%)] and *ZBTB24* [8 (5.9%)]. Accordingly, patients were allocated mostly to 5 main syndromic CID groups: syndromic DNA repair defects (n=98), HIES (n=20), congenital thrombocytopenia (n=13), EDA-ID (n=1), and others syndromic CIDs (n=5). However, we could not find any patient with confirmed monogenic forms of defects of vitamin B12 and folate metabolism, IODs and thymic defects in our cohort.

Mutations of the *ATM* were either homozygous or compound heterozygous, consisting of 51 unique variants: 25 point-mutations (10 missenses, 9 nonsenses, and 6 splicing), 20 deletions (including 10 exon deletions), 3 insertions, and 3 duplications. In other frequently mutated genes, the type of mutations was the heterozygous deletion or point mutations in the *STAT3*, homozygous point mutations in the *DNMT3B*, and hemizygous point or deletion mutations in the *WAS*. Patients with WAS and ATM deficiencies showed slightly lower survival rate compared to other main monogenic syndromic CIDs (**Figure S2**).

### Clinical manifestations

Autoimmune disorders were diagnosed in 24 (17.9%) patients at a median age of 3.5 (2.6-6.0) years, 70.6% of which were diagnosed prior to the diagnosis of immunodeficiency. The patients with autoimmunity had a shorter diagnostic delay [2.0 (0.1-4.0) vs. 3.0 (1.0-5.0); *p=0.251*]. However, the ages of onset, immunodeficiency diagnosis, and diagnostic delay were not significantly different between the patients with or without autoimmunity (*p>0.05*). Additionally, the mortality rate was lower in patients with autoimmune disorders [4 (19%) vs. 26 (24.8%); *p=0.575*].

**Figure 2:**
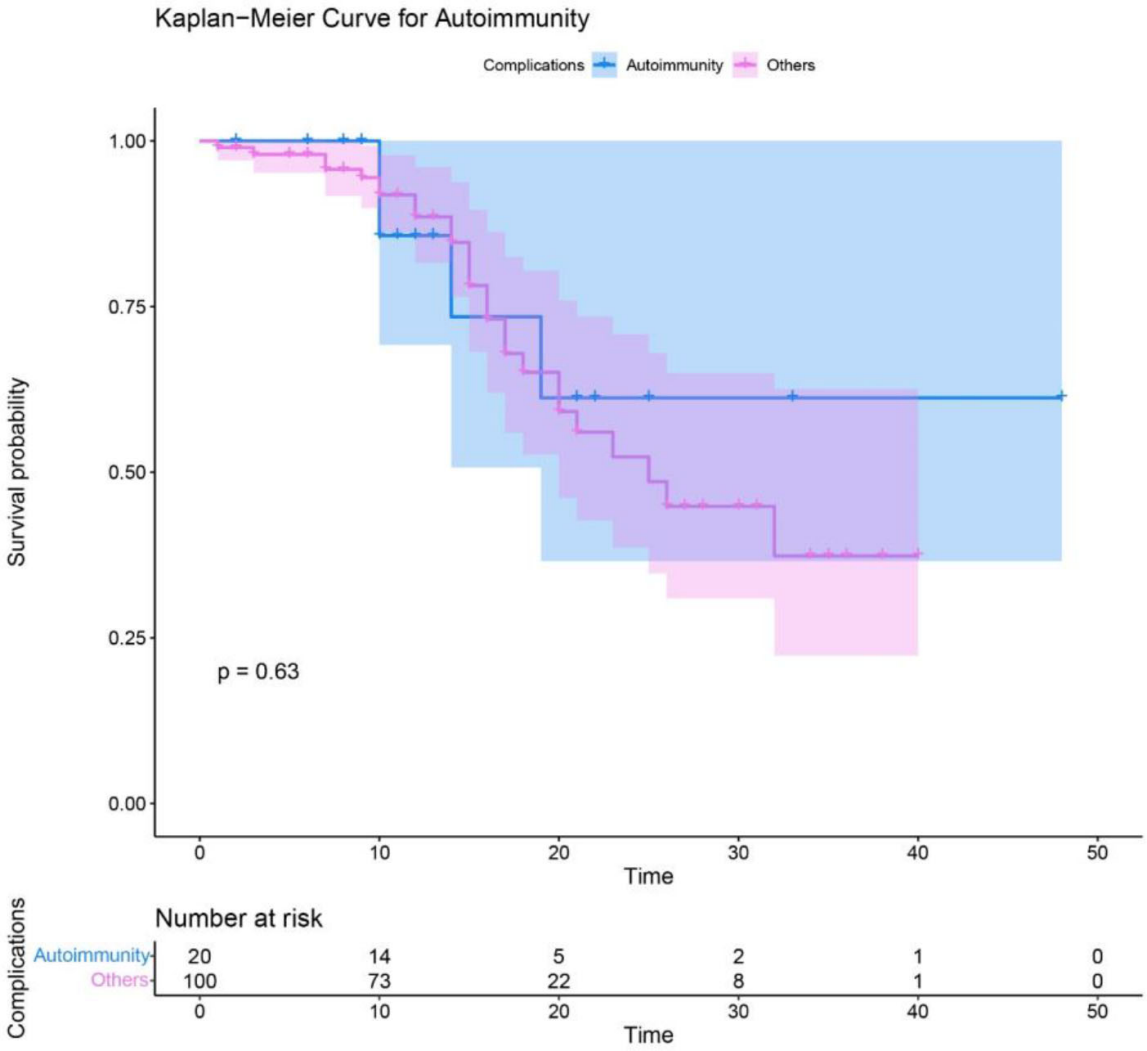
Survival analysis of syndromic CID patients with autoimmune complications compared to other patients without autoimmunity.

Autoimmune disorders appeared in 24 (17.9%) cases including immune thrombocytopenia (ITP) in 12 (50%), autoimmune hemolytic anemia (AIHA) in 4 (16.6%), rheumatoid arthritis (RA) in 4 (16.6%), inflammatory bowel disease (IBD) in 4 (16.6%), and autoimmune hepatitis (AIH) in 2 (8.3%) patients. In addition, alopecia, psoriasis, systemic lupus erythematosus (SLE), and autoimmune enteropathy were each detected in one patient (**Table 2**). The frequency of autoimmune disorders in each diagnostic group was: 22.2% with *DNMT3B/ZBTB24* [4 cases (ITP, RA, IBD)], 20% with *WAS* [2 cases (ITP)], 16.5% of patients with *ATM* [13 cases (ITP, AIHA, RA, IBD, psoriasis, SLE, AIH)], 15.8% with *STAT3 AD (LOF)* [3 cases (ITP, alopecia)], and 2 cases with *PNP* (AIH) and *TTC7A* mutations (autoimmune enteropathy). Polyautoimmunity was observed in 4 (of 134, 3%) cases [3 with *ATM* and one with *DNMT3B* gene mutations]. Of note, patients with autoimmunity have the same survival rate compared to patients without this complication (**Figure 2**).

**Table 2:** The autoimmune manifestations of Iranian patients with syndromic CID.

Parameters	Total (N=137)	ATM(N=80)	STAT3-AD (N=19)	DNMT3B/ZBTB24 (N=19)	WAS (N=11)	*^§^* P-value
Autoimmunity, n (%), (n=134)	24 (17.9%)	13 (16.4%)	3 (15.7%)	4 (22.2%)	2 (20%)	0.884
Polyautoimmunity, n (%), (n=134)	4 (3%)	3 (3.7%)	0	1(5.5%)	0	0.700
Immune thrombocytopenia, n (%),	12 (9%)	6 (7.6%)	2 (10.5%)	2 (11.1%)	2 (20%)	0.465
Autoimmune hemolytic anemia, n (%), (n=134)	4 (3%)	4 (5.1%)	0	0	0	1.000
Autoimmune enteropathy, n (%), (n=134)	1 (0.7%)	0	0	0	0	–
Juvenile idiopathic arthritis, n (%), (n=134)	4 (3%)	2 (2.5%)	0	2 (11.1%)	0	0.207
Alopecia areata, n (%), (n=134)	1 (0.7%)	0	1 (5.3%)	0	0	0.373
Systemic lupus erythematous, n (%), (n=134)	1 (0.7%)	1 (1.3%)	0	0	0	1.000
Psoriasis, n (%), (n=134)	1 (0.7%)	1 (1.3%)	0	0	0	1.000
Autoimmune hepatitis, n (%), (n=134)	2 (1.5%)	1 (1.3%)	0	0	0	1.000
Inflammatory bowel diseases, n (%), (n=134)	4 (3%)	3 (3.8%)	0	1 (5.5%)	0	0.700

n, number of patients with available data; N, total number of patients; ATM, Ataxia-telangiectasia.

*p-value is statistically significant <0.05.

§comparing ataxia-telangiectasia, HIES, WAS, and ICF syndrome (DNMT3B/ZBTB24 deficiencies).

Lymphadenopathy and hepatosplenomegaly were detected in 7 (5.2%) and 14 (10.4%) patients, respectively. Six cases (4.5%) suffered from malignancy, all in patients with *ATM*. Infectious manifestations were reported in 109 (81.3%) patients in types of otitis (43.1%), sinusitis (20.1%), candidiasis (10%), conjunctivitis (12.8%), and skin infection (33%). Severe or life-threatening infections such as bronchiectasis (10.1%) septic arthritis (4.5%), pneumonia (58.7%), sepsis (2.7%), and meningitis (2.7%) occurred in minority of patients. Other documented manifestations were as follows: failure to thrive (FTT) in 18 (13.4%), enteropathy in 16 (12%), and allergy (eczematoid rash) in 11 (8.2%) patients. Hepatosplenomegaly (*p=0.004*) and otitis (*p=0.031*) were significantly more common in syndromic patients with autoimmunity compared to patients without autoimmunity (**Table 3**), whereas the prevalence of other clinical manifestations was not significantly different in the presence or absence of autoimmune disorders (*p>0.05*).

**Table 3:** The clinical manifestations of Iranian patients with syndromic CID.

Parameters	Total (N=137)	Patients with autoimmunity (N=24)	Patients without autoimmunity (N=110)	*^†^* P-value	ATM(N=80)	STAT3-AD (N=19)	DNMT3B/ZBTB24 (N=19)	WAS (N=11)	*^§^* P-value
Infectious manifestations, n (%), (n=134)	109 (81.3%)	21 (87.3%)	88 (80%)	0.565	61 (77.2%)	18 (94.7%)	17 (94.4%)	6 (60%)	**0.042**
Otitis, n (%), (n=134)	47 (35.1%)	13 (54.2%)	34 (30.9)	**0.031**	31 (39.2%)	6 (31.6%)	7 (38.9%)	2 (20%)	0.686
Sinusitis, n (%), (n=134)	22 (16.4%)	6 (25%)	16 (14.5%)	0.229	12 (15.2%)	1 (5.3%)	7 (38.9%)	0	**0.021**
Pneumonia, n (%), (n=135)	64 (47.4%)	13 (54.2%)	50 (45.5)	0.438	31 (39.2%)	11 (57.9%)	14 (77.8%)	5 (45.5%)	**0.021**
Meningitis, n (%), (n=134)	3 (2.2%)	1 (4.2%)	2 (1.8%)	0.450	2 (2.5%)	0	0	1 (10%)	0.407
Septic arthritis, n (%), (n=135)	5 (3.7%)	1 (4.2%)	3 (2.7%)	0.550	2 (2.5%)	1 (5.3%)	0	2 (18.2%)	0.115
Skin infection, n (%), (n=135)	36 (26.7%)	6 (25%)	29 (26.4%)	0.890	16 (20.3%)	12 (63.2%)	0	7 (63.6%)	**<0.001**
Candidiasis, n (%), (n=134)	11 (8.2%)	3 (12.5%)	8 (7.3%)	0.414	3 (3.8%)	5 (26.3%)	1 (5.6%)	2 (20%)	**0.009**
Septicemia, n (%), (n=134)	3 (2.2%)	2 (8.3%)	1 (0.9%)	0.083	2 (2.5%)	1 (5.3%)	0	0	0.757
Bronchiectasis, n (%), (n=134)	11 (8.2%)	3 (12.5%)	8 (7.3%)	0.414	3 (3.8%)	4 (21.1%)	3 (16.6%)	0	**0.028**
Conjunctivitis, n (%), (n=134)	14 (10.4%)	3 (12.5%)	11 (10%)	0.716	7 (8.9%)	4 (21.1%)	3 (16.7%)	0	0.279
Lymphoproliferative involvements									
Malignancy, n (%), (n=134)	6 (4.5%)	1 (4.2%)	5 (4.5%)	1.000	6 (7.6%)	0	0	0	0.668
Hepatosplenomegaly, n (%), (n=134)	14 (10.4%)	7 (29.2%)	7 (6.4%)	**0.004**	9 (11.4%)	1 (5.3%)	3 (16.7%)	1 (10%)	0.790
Lymphadenopathy, n (%), (n=134)	7 (5.2%)	3 (12.5%)	4 (3.6%)	0.109	5 (6.3%)	2 (10.5%)	0	0	0.521
Others									
Allergy/Asthma, n (%), (n=134)	11 (8.2%)	1 (4.2%)	10 (9.1%)	0.688	7/79 (8.9%)	3 (15.8%)	1 (5.6%)	0	0.686
Neutropenia, n (%), (n=135)	13 (9.6%)	4 (16.7%)	9 (8.2%)	0.249	5/79 (6.3%)	1 (5.3%)	3 (16.7%)	3 (27.3%)	0.071
Failure to thrive, n (%), (n=134)	18 (13.4%)	6 (25%)	12 (10.9%)	0.094	10/79 (12.6%)	2 (10.5%)	5 (27.8%)	0	0.237
Clubbing, n (%), (n=134)	6 (4.5%)	1 (4.2%)	5 (4.5%)	1.000	3/79 (3.8%)	2 (10.5%)	1 (5.6%)	0	0.450

n, number of patients with available data; N, total number of patients; ATM, Ataxia-telangiectasia.

*p-value is statistically significant <0.05 (represented in bold).

†comparing patients with or without autoimmunity.

§comparing ataxia-telangiectasia, HIES, WAS, and ICF syndrome (DNMT3B/ZBTB24 deficiencies).

### Immunologic findings

In most cases, the lymphocytes counts were normal to high (84.3%). For the majority of patients, the absolute counts lymphocyte subsets were within normal range for age: CD3+ [normal (71.1%), high (21.6%), low (7.2%)], CD4+ [normal (64.3%), high (2%), low (33.7%)], CD8+ [normal (68.4%), high (24.5%), low (7.1%)], CD19+ [normal (58.9%), high (7.8%), low (33.3%)], and NK [normal (52.8%), high (25%), low (22.2%)]. Except for the predominance of low IgA (75 of 124, 60.5%), the immunoglobulins were mainly normal [normal IgG; (47.6%), normal IgM; (43.1%)]. The quality and number of lymphocytes or any lymphocyte subsets were not remarkably different in patients with or without autoimmune disorders (p>0.05). Of note, syndromic patients with autoimmunity had significantly lower IgG level (*p=0.003*). **Table 4** represents the detailed quantitative variables.

**Table 4:** The immunologic data of Iranian patients with syndromic CID.

Parameters	Total (N=137)	Patients with autoimmunity (N=24)	Patients without autoimmunity (N=110)	*^†^* P-value	ATM(N=80)	STAT3-AD (N=19)	DNMT3B/ZBTB24 (N=19)	WAS (N=11)	*^§^* P-value
WBC, (cell/μL), median (IQR), (n=122)	6940 (4930-10625)	5690 (3302-9850)	7120 (5020-10700)	0.213	5680 (4595-8410)	13350 (8430-18400)	6900 (4930-10475)	8630 (5995-16640)	**<0.001**
ALC, (cell/μL), median (IQR), (n=121)	2151 (1453-3749)	2109 (1388.4-3294.6)	2187.9 (1451.2-3845)	0.560	1775 (1188-2522)	5520 (2696-6912)	2821 (1849-4979)	2108.7 (1594.4-3958.5)	**<0.001**
Lymphocytes, (%), median (IQR), (n=121)	33 (23.4-47)	33 (21.6-54.5)	33 (23.9-46.3)	0.546	31 (21.6-41.5)	30 (24.5-53.7)	40 (32-52)	20 (11.5-48.1)	**0.026**
ANC, (cell/μL), median (IQR), (n=116)	3425 (2211-5622)	3021 (1210-4303)	3581.5 (2257.5-5782.2)	0.154	3094 (2180-5139)	5442 (3284-11333)	2774 (1814-4848)	4979 (2917-7398)	**0.046**
Neutrophils, (%), median (IQR), (n=116)	51.7 (39.7-61.9)	46 (31.5-61)	52 (40.7-63.5)	0.287	54.3 (43.7-67.2)	51.5 (30-67.2)	44 (35-53.3)	52 (35.2-63.8)	0.087
CD3+ T cells (%), median (IQR), (n=97)	64.8 (49.6-79)	69 (53-79)	62.7 (48.1-79)	0.337	56.8 (42-72.1)	70.4 (65.3-79.7)	74.5 (64.2-81)	60 (50.6-83.5)	**0.006**
CD4+ T cells (%), median (IQR), (n=98)	33.7 (21.5-41.2)	36.7 (26.9-42)	33.4 (19.1-41)	0.358	27.5 (17.2-38.5)	40 (35.1-45)	39 (27.5-43.5)	30.7 (23-42)	**0.007**
CD8+ T cells (%), median (IQR), (n=98)	25.5 (19.4-35.2)	24 (18.8-30.2)	26 (19.4-36)	0.653	22.9 (18.7-37.7)	28 (20.7-34.2)	30 (21-37)	29 (9-41)	0.674
NK cell, (%), median (IQR), (n=37)	7 (2.5-23)	4 (2-14.3)	9 (4-29.2)	0.108	8 (1.4-23)	6 (5-7)	14.3 (7.5-36.5)	41 (2-)	0.579
CD19+ B cells (%), Median (IQR), (n=92)	13 (5.1-19)	9.2 (2.4-19.7)	13 (6.6-19)	0.392	10 (4.3-16.1)	15 (7-22.7)	11.5 (5.5-22.2)	18 (4-27.5)	0.394
IgM, (mg/dl), median (IQR), (n=124)	122.5 (42.2-235.2)	143.5 (30.7-593.5)	122.5 (51.2-217.2)	0.464	196 (104.7-328.7)	129 (95-175)	24 (5-56.5)	25 (9-31)	**<0.001**
IgA, (mg/dl), median (IQR), (n=125)	11 (3.7-68)	10 (2-54.7)	11 (4-75)	0.861	7 (2-33)	129 (66-230)	8.5 (1-31.5)	28 (5-140)	**<0.001**
IgG, (mg/dl), median (IQR), (n=125)	687 (230-1063)	220 (57.5-794.5)	771 (365-1133)	**0.003**	687 (280-957)	1450 (941-1600)	306 (53.2-644.2)	645 (190-898)	**<0.001**
IgE, (mg/dl), median (IQR), (n=115)	3 (1-21)	1 (0-8.1)	3.2 (1-58)	0.063	1 (1-5)	1642 (853-9770)	4 (0-6.5)	36.5 (2.7-140.2)	**<0.001**

N, number of patients with available data; N, total number of patients; ATM, Ataxia-telangiectasia; WBC, White blood cells; ALC, Absolute lymphocytic count; ANC, Absolute neutrophilic count; NK, Natural killer; Ig, Immunoglobulin.

The median is shown [with 25th and 75th percentiles].

*p-value is statistically significant <0.05 (represented in bold).

†comparing patients with or without autoimmunity.

§comparing ataxia-telangiectasia, WAS, HIES, and ICF syndrome (DNMT3B/ZBTB24 deficiencies).

### The comparison of ATM, STAT3 AD (LOF), DNMT3B/ZBTB24, WAS mutations

Considering the majority of our patients were categorized into *ATM*, *STAT3 (AD-LOF)*, *DNMT3B/ZBTB24*, and *WAS* mutations, we compared these mutation groups. Patients with *ATM* had the latest disease onset with a median (IQR) of 12.0 (11.0-24.0) months [vs. *STAT3 (AD-LOF*) with 0.2 (0.1-3.0), *DNMT3B/ZBTB24* with 3.0 (0-6.0), and *WAS* with 0.1 (0-0.5); *p<0.001*]. Cases with *DNMT3B/ZBTB24* mutations were the younger at the time of diagnosis with 2.0 (0.7-4.5) years [vs. *STAT3 AD (LOF)* with 6.0 (3.0-12.0); *p=0.019*]. The patients with *ATM* predominantly presented ataxia/telangiectasia (46.2%) and infections (32.1%), while allergy (52.6%) and infection (94.4%) were the dominant first manifestations in *STAT3 (AD-LOF)* and *DNMT3B/ZBTB24* mutations, respectively. Also, cases with *WAS* mutation were primarily manifested infection (44.4%). Most patients in the *ATM* (92.5%) and *STAT3 (AD-LOF)* (89.5%) groups had similar clinical diagnoses, however, patients with *DNMT3B/ZBTB24* mutations were first diagnosed with hypogammaglobulinemia (13, 68.4%), immunoglobulin A deficiency (2, 10.5%), and agammaglobulinemia (1, 5.3%). Hyper IgM syndrome (3, 3.8%), hypogammaglobulinemia (1, 1.3%), and immunoglobulin A deficiency (1, 1.3%) were the other first diagnoses in selected AT patients. Although patients with *DNMT3B/ZBTB24* variants had the longest time of follow-up [24.0 (12.0-33.0) years; *p<0.001*], they had the lowest mortality rate (*p=0.013*).

Neither was the prevalence of autoimmune manifestation nor any of its subtypes statistically significant between the groups (**Table 2**). In addition to the overall infection rate, the following types of infection were markedly different in the comparison: sinusitis [38.9% in *DNMT3B/ZBTB24* mutations; *p=0.021*], pneumonia [77.8% in *DNMT3B/ZBTB24* mutations; *p=0.021*], candidiasis [26.3% in *STAT3 (AD-LOF)* mutation; *p=0.009*], cutaneous infection [63.2% in *STAT3 (AD-LOF)* mutation; *p<0.001*], bronchiectasis [21.1% in *STAT3 (AD-LOF)* and 16.7% in *DNMT3B/ZBTB24* mutations; *p=0.028*].

Lymphocyte and CD3+ cells frequencies were the lowest in patients with ATM deficiency compared to the *DNMT3B/ZBTB24, WAS*, and *STAT3 (AD-LOF)* mutations (*p<0.001* and *p=0.046*, respectively). IgG, IgA, and IgE levels were higher than the normal range in the *STAT3 (AD-LOF)* mutation, whereas, patients with ATM deficiency had the highest level of IgM (**Table 4**). Low levels of IgA, IgM, and IgG were the common immunoglobulin dysregulations that occurred in 61.1%, 66.7%, and 55.6% of individuals with *DNMT3B/ZBTB24* mutations, respectively. Of the 27 cases with abnormal IgE levels, 19 cases had *STAT3 (AD-LOF)* mutation and this group had the highest amount of serum IgE (**Table 4**). Patients with *ATM* variation mostly had a high level of IgM (37 cases, 50%), normal levels of IgG (40 cases, 53.3%) and IgE (65 cases, 94.2%), and low level of IgA (56 cases, 74.7%). *WAS*-mutated patients mainly had low IgM (4 cases, 57.1%) and normal to high levels of IgE, IgA, and IgG.

## Discussion

In this study, we evaluated the clinical, immunological, and genetic features of 137 Iranian patients with syndromic CIDs, mainly diagnosed with *ATM*, *DNMT3B/ZBTB24*, *WAS* and *STAT3 AD (LOF)* variants. More than 95% of our patients presented the initial symptoms from birth to early childhood (up to 5 years old). Consanguinity was present in about 77% of our patients, similar to a prevailing study of Iranian CID patients (16). Considering skin manifestations usually appear within a few weeks after birth in patients with STAT3 deficiency (17) and parallel with Tavassoli et al. (18), eczematoid lesions were the earliest manifestation in more than half of our patients with *STAT3 AD (LOF)* mutation.

CID patients with syndromic features develop a broad range of clinical manifestations, particularly autoimmune involvements (19). Autoimmune manifestations, especially hematological, occurred in 17.9% of our studied population. Although there was no significant difference among the studied groups, autoimmunity was a prevalent finding in *DNMT3B/ZBTB24* (22.2%) and *WAS* mutations (20%). Similarly, previous studies have reported a high prevalence of autoimmune disorders among WAS patients (20), but also introduced it as a rare manifestation in mutated *DNMT3B/ZBTB24* (21). Interestingly, these autoimmune manifestations were diagnosed before immunodeficiency in more than two-thirds of the affected patients and even five cases first manifested with autoimmunity. The presence of autoimmunity in syndromic CID patients was remarkably associated with hepatosplenomegaly and lower level of IgG, which might be justified by the presence of autoantibodies in ITP as the main autoimmunity of our patients (22). Taken together, autoimmunity, especially hematological autoimmune diseases, could be considered as an early-onset involvement with a potential diagnostic role in suspicious cases.

The possible mechanisms of autoimmunities in ATM deficiency were introduced either secondary to immunodeficiency or as an effect of the lack of ATM protein (5, 23, 24). The absence or decreased expression of WAS protein and the subsequent immune impairments might be responsible for autoimmune complications in patients with *WAS* mutation (20). Altered phosphorylation of STAT3 in STAT3 deficiency (25) or DNA methylation in *DNMT3B* and *ZBTB24* are probably accounted for the autoimmunity in these diseases (26).

Despite being insignificant, our patients with autoimmunity were inclined to have a shorter diagnostic delay and lower mortality rate. This outcome could arise from the increased awareness and medical follow-up of autoimmune manifestations in the last decades, especially after introducing autoimmunity as a warning sign for primary immunodeficiency (27). Totally, in addition to the overall infection rate, distinct types of infection including sinusitis, pneumonia, candidiasis, cutaneous infection, and bronchiectasis were considerably different among the four groups. Since STAT3 activation is essential for T helper (Th)17 cell proliferation and consecutive IL-17 secretion (28), the marked increase of candidiasis in the STAT3 (AD-LOF) is attributed to the impaired IL-17 immunity in these patients (29). Also, considering eczema is a risk factor for cutaneous infection (30), this type of infection was frequently detected in patients with mutated *STAT3 (AD-LOF)* and *WAS*. Furthermore, the higher frequency of sinusitis in patients with *DNMT3B/ZBTB24* mutations might result from facial dysmorphisms associated with severe antibody production impairment. Thus, the type of infections might be beneficial in determining the category of syndromic CIDs.

Compatible with the immunologic features available in IUIS 2019 (2), hyper IgE and hypogammaglobulinemia was found in the majority of patients with *STAT3 AD (LOF)* and *DNMT3B/ZBTB24* mutations, respectively. In addition, patients with *ATM* variants were associated with hyper IgM and hypo IgA serum levels, the same Ig dysregulations that are anticipated in these patients. Hypo IgM was also commonly detected in Wiskott-Aldrich syndrome (2). More than 18% of all patients with syndromic CIDs, including most cases with *DNMT3B/ZBTB24* mutations, were clinically diagnosed with antibody deficiencies before genetic evaluation. It possibly stems from the shared immunologic features of hypogammaglobulinemia and normal B cell counts in the absence of remarkable facial abnormalities in our *DNMT3B/ZBTB24*-mutated patients. Comparable to former reports (31, 32), a few patients with ATM deficiency were initially classified as HIgM and IgAD. Lack of neurological manifestations and close immunologic results in these patients might have led to some variants of *ATM* being misdiagnosed as HIgM and IgAD. Therefore, physicians should be aware that syndromic CIDs may resemble predominantly antibody deficiencies and should be investigated in patients with a tentative diagnosis, especially in cases presenting with incomplete features.

We had one female WAS patient who was homozygous for the mutation and later deceased. Considering the death of her parents shortly after the patient and the unavailability of the father’s clinical information and the DNA samples of the parents, it is not possible to investigate whether she was born in a consanguineous union with a WAS-mutated father be partnered with a WAS carrier. Although it may seem like only about 20% of the syndromic CIDs (11 out of 60 causative genes) are addressed in this study, the mentioned categories, especially *ATM* and *DNMT3B/ZBTB24*, are the most common disorders identified in previous reports of CIDs with associated or syndromic features (11, 33, 34). Noteworthily, more than half of our patients are ATM deficient, and the frequency is quite skewed, which is different from worldwide frequency. Therefore, future studies should be conducted in various country registrations.

## Conclusions

In conclusion, almost one-fifth of Iranian patients with syndromic CID developed autoimmunity, mainly in the form of hematological immune diseases. Autoimmunity could be considered as an early-onset involvement with a potential diagnostic role in suspicious cases of syndromic CID. The immunologic data of the patients were compatible with the expected features in each diagnostic category however, no genotype-phenotype correlation was identified. More than 18% of all patients with syndromic CIDs, especially most of our patients with *DNMT3B/ZBTB24* mutations, were initially misdiagnosed as predominantly antibody deficiencies before genetic evaluation. Therefore, patients with a tentative diagnosis should be examined more carefully, especially in cases presenting with incomplete features.

## Data availability statement

The datasets presented in this article are not readily available because of the informed consent obtained from the patients/parents based on the ethic committee permission. Requests to access the datasets should be directed to the corresponding authors.

## Ethics statement

This study was approved by the Ethics Committee of the National Institute for Medical Research Development (IR.NIMAD.REC.1400.086). Prior to data collection, written informed consent has been obtained from each patient and/or their parents.

## Author contributions

All authors contributed to the study conception and design. Material preparation, data collection and analysis were performed by NS, GA, ZC, SM, AS, AV, MS, FA, FN SD, MJ, FF, PS, RM, ME, RY, NR, and HA. The first draft of the manuscript was written by NS and all authors commented on previous versions of the manuscript. All authors contributed to the article and approved the submitted version.

## Funding

Research reported in this publication was supported by Elite Researcher Grant Committee under award number [4000096] from the National Institute for Medical Research Development (NIMAD), Tehran, Iran.

## Conflict of interest

The authors declare that the research was conducted in the absence of any commercial or financial relationships that could be construed as a potential conflict of interest.

## Publisher’s note

All claims expressed in this article are solely those of the authors and do not necessarily represent those of their affiliated organizations, or those of the publisher, the editors and the reviewers. Any product that may be evaluated in this article, or claim that may be made by its manufacturer, is not guaranteed or endorsed by the publisher.
