# Supplementary material for: Autoimmunity in monogenic combined immune deficiencies with associated or syndromic features
Source: Front Immunol. 2022 Dec 5;13:1023127. doi: 10.3389/fimmu.2022.1023127 (PMC9760934; doi:10.3389/fimmu.2022.1023127)
Supplement: Supplementary file 1 [file DataSheet_1.docx]

**Supplementary Data**

**Journal of Frontiers in Immunology**

**Autoimmunity in monogenic combined immune deficiencies with associated or syndromic features**

Niusha SHARIFINEJAD^1^, Gholamreza AZIZI^1,2*^, Zahra CHAVOSHZADEH^3^, Seyed Alireza MAHDAVIANI^4^, Mahnaz SEIFI ALAN^5^, [Marzieh TAVAKOL](https://pubmed.ncbi.nlm.nih.gov/?sort=date&size=100&term=Tavakol+M&cauthor_id=33774840)^1^, [Homa SADRI](https://pubmed.ncbi.nlm.nih.gov/?sort=date&size=100&term=Sadri+H&cauthor_id=31456526)^1^, Mohammad NABAVI^6^, Sareh Sadat EBRAHIMI^7^, Afshin SHIRKANI^8^, Ahmad VOSUGHI MOTLAGH^9^, Molood SAFARIRAD^9^, Fatemeh AGHAMAHDI^1^, Farzad NAZARI^2^, [Samaneh DELAVARI](https://pubmed.ncbi.nlm.nih.gov/?sort=date&size=100&term=Delavari+S&cauthor_id=33774840)^2^, [Mahnaz JAMEE](https://pubmed.ncbi.nlm.nih.gov/?sort=date&size=100&term=Jamee+M&cauthor_id=33774840)^10^, Farimah FAYYAZ^11^, Parham SAMIMISEDEH^12^, Rahman MATANI^1^, Marzie ESMAEILI^2^, [Reza YAZDANI](https://pubmed.ncbi.nlm.nih.gov/?sort=date&size=100&term=Yazdani+R&cauthor_id=33774840)^2^, Nima REZAEI^2^, Hassan ABOLHASSANI^2,13*^

1. Non-communicable Diseases Research Center, Alborz University of Medical Sciences, Karaj, Iran.
2. Research Center for Immunodeficiencies, Pediatrics Center of Excellence, Children's Medical Center, Tehran University of Medical Sciences, Tehran, Iran.
3. Pediatric Infections Research Center, Mofid Children's Hospital, Shahid Beheshti University of Medical Sciences, Tehran, Iran.
4. Pediatric Respiratory Diseases Research Center, National Research Institute of Tuberculosis and Lung Diseases, Shahid Beheshti University of Medical Sciences, Tehran, Iran.
5. Cardiovascular Research Center, Alborz University of Medical Sciences, Karaj, Iran.
6. Department of Allergy and Clinical Immunology, Rasool e Akram Hospital, Iran University of Medical Sciences, Tehran, Iran.
7. Department of Immunology and Allergy, Kerman University of Medical Sciences, Kerman, Iran.
8. Allergy and Clinical Immunology Department, School of Medicine, Bushehr University of Medical Science, Moallem St., Bushehr, Iran.
9. Department of Pediatrics, North Khorasan University of Medical Sciences, Bojnurd, Iran.
10. Pediatric Nephrology Research Center, Research Institute for Children's Health, Shahid Beheshti University of Medical Sciences, Tehran, Iran.
11. Cancer Immunology Project (CIP), Universal Scientific Education and Research Network (USERN), Tehran, Iran.
12. Cardiovascular Research Center, Alborz University of Medical Sciences, Karaj, Iran.
13. Division of Clinical Immunology, Department of Biosciences and Nutrition, Karolinska Institutet, Karolinska University Hospital, Huddinge, Stockholm, Sweden.

**Corresponding authors:**

Hassan Abolhassani, MD, MPH, PhD.

Division of Clinical Immunology, Department of Biosciences and Nutrition, NEO, Blickagangen 16, Karolinska Institute, Stockholm, SE-14157, Sweden.

Tel: +46 8 5248 1117

Email: [hassan.abolhassani@ki.se](mailto:hassan.abolhassani@ki.se)

Gholamreza Azizi, PhD.

Non-communicable Diseases Research Center, Vice Chancellor for Research, Alborz University of Medical Sciences, Karaj, Iran.

Tel: +98 9123665512

Email: [azizi@abzums.ac.ir](mailto:azizi@abzums.ac.ir)

**Table S1. DNA variants identified in genes related to CID with syndromic features.**

| **Gene symbol** | **Nucleotide change (Protein change)** | **Mutation type** | **Inheritance** (**Zygosity)** |
| --- | --- | --- | --- |
| *ATM* (n=80) | **c. 8741T>A**(p.Ile2914Asn), **c.1159A>C**(p.Lys387Gln), **c.8741T>A**(p.Ile2914Asn), **c.5003T>G** (p.Leu1668Arg), **c.6047A>G** (p.Asp2016Gly), **c.6452G>C** (p.Arg2151Thr), **c.6744G>A** (p.Lys2248=), **c.7788G>A** (p.Glu2596=), **c.7865C>T** (p.Ala2622Val). | Point Mutation (Mis) | AR (Hom) |
|  | **c.829G>T**(p.Glu277Ter), **c.6658C>T**(p.Gln2220Ter), **c.1537C>T** (p.Gln513Ter), **c.8050C>T**(p.Gln2684Ter), **c.664C>T** (p.Gln222Ter), **c.6658C>T** (p.Gln2220Ter), **c.67C>T** (p.Arg23Ter), **c.829G>T** (p.Glu277Ter), **c.4864G>T** (p.Glu1622Ter). | Point Mutation (Non) |  |
|  | **c.6199-1G>T**,**c.6807+1G>C**, **c.2921+1G>T**, **c.6453-2A>G**, **c.6453-2A>G**, **c.7308-6T>G**. | Point Mutation (Spl) |  |
|  | **c.3244_3245insG** (p.His1082ArgfsTer14), **c.5552_5553insC** (p.Gln1852ProfsTer5), **c.8375_8376insC** (p.Arg2792SerfsTer4), **c.3600_3601delTT** (p.Phe1201TrpfsTer3), **c.3895delG** (p.Ala1299ProfsTer50), **c.5585delT**(p.Ser1863LeufsTer54), **c.634delT** (p.Ser214ProfsTer16), **c.8046-8047delTA** (p.Ile2683ThrfsTer4), **c.7883delT** (p.Ile2629TyrfsTer2). | Frameshift Mutation |  |
|  | **del EX61-EX62**, **del EX61-EX63**, **del EX61-EX64**, **del EX61-EX65**, **del EX62-EX63**, **del EX37-EX48**, **del EX59-EX60**, **Del EX1**. | Large Deletion |  |
|  | **c.5712dupA** (p.Ser1905IlefsTer25), **c.9097_9101dupAATTT**(p.Leu3035IlefsTer8), **c.9097_9101dupAATTT**(p.Leu3035IlefsTer8). | Duplication |  |
|  | **c.6259delG**(p.Glu2087LysfsTer9) and **c.6658C>T** (p.Gln2220Ter), **c.8907T>G**(p.Tyr2969Tyr) and **c.8050C>T**(p.Gln2684Ter), **c.8907T>C**(p.Tyr2969Tyr) and **c.2639-1G>A**, **dup EX18-EX61** and **c.7788G>A** (p.Glu2596=), **c.7883delT** (p.Ile2629TyrfsTer2) and **c.7668delT** (p.Leu2557CysfsTer7), **c.7883delT**(p.Ile2629TyrfsTer2) and **c.7655delA** (p.His2552ProfsTer12), **c.8268G>C**(p.Lys2756Asn) and **c.7883delT**(p.Ile2629TyrfsTer2), **c.8280delC**(p.Ser2761LeufsTer45) and **c.7883delT**(p.Ile2629TyrfsTer2). | - | AR (CH) |
| *STAT3* (n=19) | **c.1144C>T** (p.Arg382Trp), **c.1145G>A** (p.Arg382Gln), **c.1909G>A** (p.Val637Met), **c.2147C>T** (p.Thr716Met), **c.23C>T** (p.Ala8Val), **c.1863C>G** (p.Phe621Leu) | Point Mutation (Mis) | AD (Het) |
|  | **c.1479delT**(p.Phe493LeufsTer16), **c.1971delT**(p.Lys659ArgfsTer28) | Frameshift Mutation |  |
| *DNMT3B* (n=11) | **c.1519G>A** (p.Ala507Thr), **c.1823G>A** (p.Arg670Gln), **c.1871A>G** (p.Glu624Gly), **c.2428G>T** (Gly810Cys), **c.2356 G>A** (p.Glu806Lys), **c.1878 T>A** (p.Ile646Ile) | Point Mutation (Mis) | AR (Hom) |
|  | **c.2397-11G>A** | Point Mutation (Spl) |  |
| *WAS* (n=11) | **c.130G>A**(p.Leu44Met), **c.397G>A** (p.Glu133Lys), **c.911G>C**(p.Arg304Pro) | Point Mutation (Mis) | XLR (Hemi) |
|  | **c.961C>T**(p.Arg321Ter) | Point Mutation (Non) |  |
|  | **c.1453 +1G>C**, **c.777+1G>A** | Point Mutation (Spl) |  |
|  | **c.687delG** (p.Lys230fs) | Frameshift Mutation |  |
| *ZBTB24* (n=8) | **c.1224C>G** (p.Cys408Trp), **c. 1148 G>C** (p.Cys383Ser), **c.2166T>A** | Point Mutation (Mis) | AR (Hom) |
|  | **c.795dupA**(p.Asp266ArgfsTer28) | Frameshift Mutation |  |
|  | **c.1371_1381delTG** (p.Arg457GlyfsTer13) | Point Mutation (Non) |  |
| *ARPC1B* (n=2) | **c.897_910del** (p.Glu300ProfsTer153) | Frameshift Mutation | AR (Hom) |
| *TTC7A* (n=2) | **c.164C>T** (p.Ala55Val) | Point Mutation (Mis) | AR (Hom) |
|  | **c.1955delA** (p.Glu652GlyfsTer7) | Frameshift Mutation |  |
| *CARD11* (n=1) | **c.1009C>T** (p.Arg337Ter) | Point Mutation (Non) | AR (Hom) |
| *IKBKG* (n=1) | **c.932A>G** (p.Asp311Gly) | Point Mutation (Mis) | XLR (Hemi) |
| *PNP* (n=1) | **c.208T>C** (p.Phe70Leu) | Point Mutation (Mis) | AR (Hom) |
| *KMT2D* (n=1) | **c.10024C>T** (p.Arg 3342Cys) | Point Mutation (Mis) | AD (Het) |
| *AD: Autosomal Dominant, AR: Autosomal Recessive, ARPC1B: Actin Related Protein 2/3 Complex Subunit 1B, ATM: ATM; Ataxia telangiectasia mutated, CARD11: Caspase Recruitment Domain family member 11, CH; Compound Heterozygous, DNMT3B: DNA Methyltransferase 3 Beta, Hem: Hemizygous, Het: Heterozygous, Hom: Homozygous, IKBKG: Inhibitor of Nuclear Factor Kappa B kinase Regulatory Subunit Gamma, Splicing: Spl, KMT2D: lysine Methyltransferase 2D, Mis: Missense, Non: Nonsense, PNP: Purine Nucleoside Phosphorylase, STAT3: Signal Transducer and Activator of Transcription 3. TTC7A: tetratricopeptide repeat domain 7A, WAS: WASP actin nucleation promoting factor, XLR: X-linked Recessive, ZBTB24: zinc finger and BTB domain containing 24.* | | | |

**
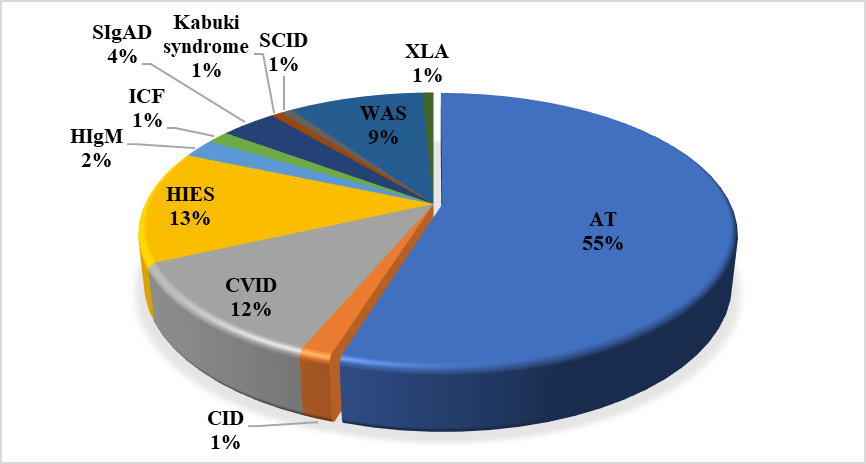
**

**Figure S1. The first clinically diagnosis in Iranian patients with syndromic CID.** AT; Ataxia-telangiectasia, CID; Combined immunodeficiency, CVID; Common variable immunodeficiency, HIES; Hyper IgE Syndrome, HIgM; Hyper IgM syndrome, ICF; Immunodeficiency with centromeric instability and facial anomalies, SIgAD; Selective IgA deficiency, SCID; Severe combined immunodeficiency, WAS; Wiskott–Aldrich syndrome, XLA; X-linked agammaglobulinemia.

**
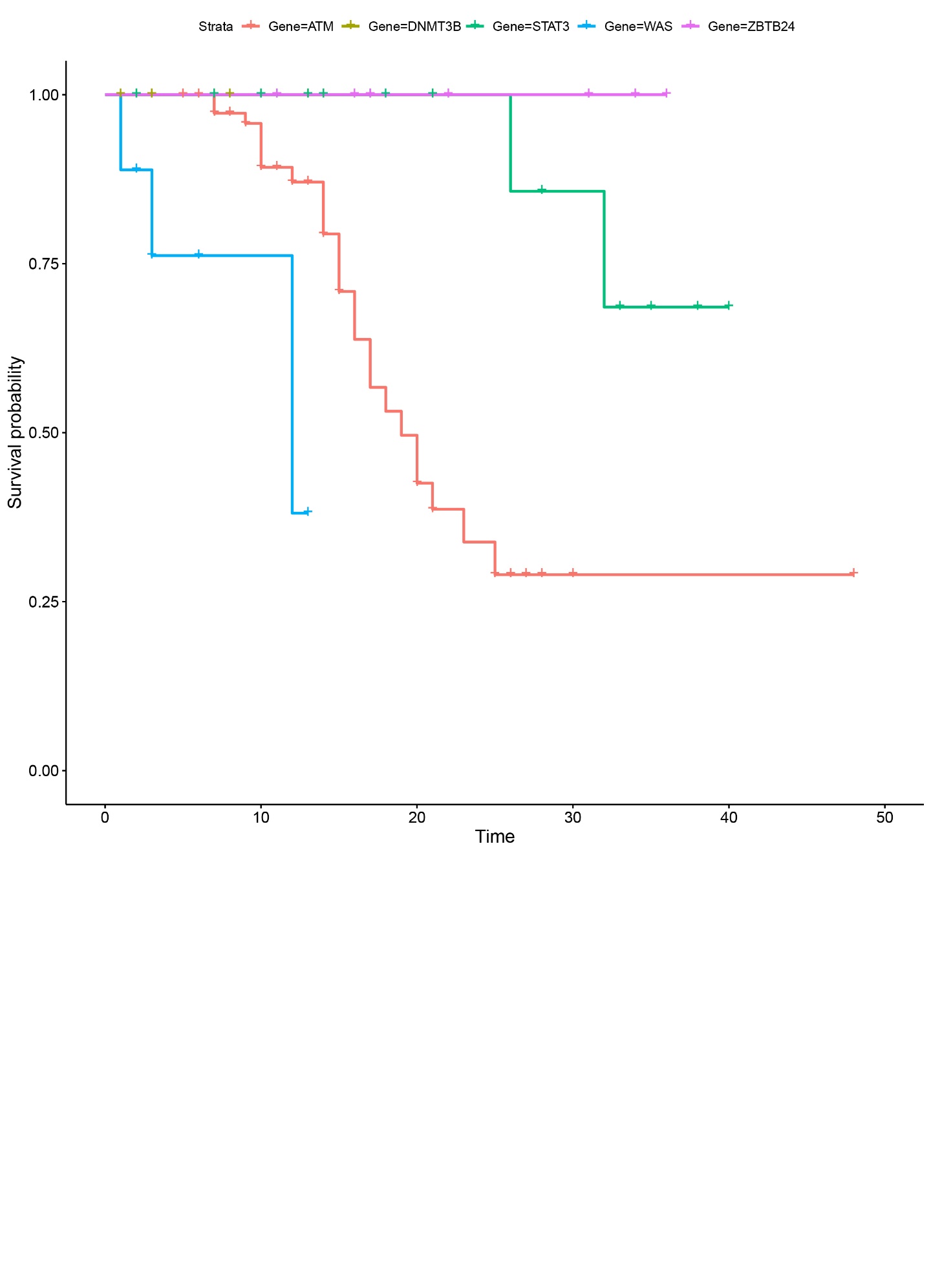
**

**Figure S2. Survival of Iranian patients with syndromic CID within most frequent genetic defects.**
